# Are midwives trained to recognise perinatal depression symptoms? Results of MAMA (MAternal Mood Assessment) cross-sectional survey in Italy

**DOI:** 10.1007/s00737-024-01439-z

**Published:** 2024-02-03

**Authors:** Claudia Ravaldi, Laura Mosconi, Giada Crescioli, Giulia Lombardo, Ilenia Russo, Angelo Morese, Valdo Ricca, Alfredo Vannacci

**Affiliations:** 1 PEARL Perinatal Research Laboratory, CiaoLapo Foundation, Department of Neurosciences, Psychology, Drug Research and Child Health, University of Florence, Viale Pieraccini 6, 50139 Florence, Italy; 2 Unit of Obstetrics and Gynecology, Parma University Hospital, Parma, Italy; 3 Unit of Obstetrics and Gynecology, “S. Marta E S. Verera” Hospital, ASP Catania, Acireale, Italy; 4 Section of Pediatrics, Obstetrics and Gynecology and Nursing, Department of Health Sciences, University of Florence, Florence, Italy; 5 Section of Psychiatry, Department of Health Sciences, University of Florence, Florence, Italy

**Keywords:** Perinatal depression, Screening, Midwifery, Maternal mental health, Trauma-informed care

## Abstract

**Purpose:**

To assess the knowledge, clinical experience, and attitudes of Italian midwives toward perinatal depression (PND) and to explore how these factors impact the quality of care.

**Methods:**

We conducted a cross-sectional online survey among 152 midwives employed in public hospitals across Italy. The questionnaire covered a range of topics, including demographic data, professional experience, knowledge of PND symptoms, risk factors, and clinical management, as well as communication skills and personal experiences with PND cases.

**Results:**

A concerning 76.3% of midwives displayed inadequate knowledge of PND based on current scientific literature. Those with a more comprehensive understanding were notably more confident in their practice, expressing significantly fewer apprehensions about communicating with mothers (25.8% vs 74.2%) and lesser concerns about the mothers’ future well-being (38.9% vs 62.95%). The survey results also emphasised the midwives’ call for specialised guidelines and formal training in PND management and underscored the value of communication skills, continuity of care, and family engagement in supporting affected mothers.

**Conclusion:**

This inaugural study sheds light on the current state of knowledge and attitudes among Italian midwives regarding PND. It pinpoints crucial areas for educational enhancement and practice improvement, suggesting that elevated levels of midwife expertise in PND could significantly elevate the standard of care and expedite early diagnosis and treatment.

## Introduction

Perinatal depression (PND) is a significant global health issue, with prevalence rates varying widely. Studies report ranges from 6.5 to 19% among pregnant and postpartum women (Gavin et al. 2005; Woody et al. 2017). In Italy, recent studies show a prevalence of 6.4% during pregnancy and approximately 20% postpartum (Cena et al. 2021). The variations in the prevalence of PND reported in international literature may stem from the use of diverse self-report assessment tools, as well as the differing clinical, sociodemographic, and economic characteristics of the study samples (Cena et al. 2021). Despite this high prevalence, PND remains largely underdiagnosed (Earls et al. 2010; Faisal-Cury et al. 2021). Contributing to this issue is the pervasive social stigma associated with mental illness, which is a barrier not only for the general public but also for healthcare professionals (HCPs) (Henderson et al. 2014; Thornicroft et al. 2016). This stigma can have a cascade of negative consequences, including underdiagnosis, barriers to accessing mental healthcare services, and a lack of social acceptance and support (Knaak et al. 2017). One primary driver of this stigma is a general lack of knowledge about mental health issues, such as attributions of incompetence and blame for the own mental health condition (Barney et al. 2006), a gap that could be bridged through targeted formal training (Thornicroft et al. 2007). For perinatal women, the stigma manifests as shame and reluctance to seek help, often fueled by the fear of being labelled as “bad mothers” (Cornally & McCarthy 2011; McLoughlin 2013). Nonetheless, mothers are subjected to several social norms, as underlined by a recent scoping review (Schmidt et al. 2023), which could be an additional burden in the journey of motherhood. Besides stigma, mothers’ inclination to seek help is shaped by two additional factors: the tendency to underestimate their symptoms and their interactions with HCPs. Therefore, it is crucial that women encounter positive experiences within the healthcare system and are adequately informed about the onset of PND (Button et al. 2017).

Formal diagnosis is an essential starting point for treating PND and international literature recommends screening for perinatal depression several times during pregnancy and postpartum period (Moore Simas et al. 2023). This process is fraught with challenges, including inconsistencies in screening tools and the inherent difficulties of the diagnostic process itself. Complicating matters are symptoms like altered sleep patterns, appetite changes, and excessive fatigue, which closely mimic natural experiences during and post-pregnancy (Boyd et al. 2005). Early identification of depression during pregnancy is crucial as it is the strongest predictor for extending into the postpartum period (Leigh & Milgrom 2008). Several well-established risk factors exist and could be grouped into three categories: biological, environmental, and psychosocial (ACOG 2023). Some examples are as follows: a previous history of depression and/or anxiety (Gaillard et al. 2014; McGrath et al. 2008; Räisänen et al. 2014), lack of social support (Eastwood et al. 2012; Yağmur & Ulukoca 2010), intrusive memories of traumatic events (Grekin et al. 2022), and exposure to stressful life events (Escribà-Agüir & Artazcoz 2011). Although a variety of psychological assessment tools are available to assist HCPs, most are not tailored specifically for PND but are designed to evaluate depressive symptoms more broadly. A notable exception is the Edinburgh Postnatal Depression scale (Cox et al. 1987) that is an easy-to-use 10-item self-report scale which was originally created for postnatal depression but was also found effective in detecting prenatal depression in multiple studies. (Park & Kim 2022). However, it has been shown that the EPDS has several limitations such as a variety of validated cut-off scores which create confusion in clinical practice (Matthey et al. 2006; Matthey & Agostini 2017).

Midwives occupy a unique position in women’s healthcare, serving as critical touchpoints for identifying mental health issues, including PND (El-Den et al. 2022; Martin et al. 2020). They have a privileged role in observing mothers from the situation of a suspected PND to the official screening assessment. A recent paper by Martin et al. (2020) points out the insufficient training of midwives about PND, leading mothers who suffered this situation to miss out on proper treatment. Italian midwives usually receive only a short training in general psychology during their undergraduate pathway, which could be insufficient to gain a good understanding of PND features (Decreto Interministeriale 2 aprile 2001—Determinazione delle classi delle lauree universitarie delle professioni sanitarie, 2001).

The lack of a proper PND-specific diagnostic tool (Martin et al. 2020) and the challenges of differentiating depressive symptoms from typical pre and postpartum experiences add layers of complexity (Boyd et al. 2005). Moreover, literature underlines that midwives think to be ill-equipped on perinatal mental health knowledge and they need appropriate education about this topic (Hauck et al. 2015; Mccauley et al. 2011; Noonan et al. 2017). While the literature broadly advocates for universal PND screening, there is no consensus on the most effective and specific instruments for this purpose (El-Den et al. 2022). Identifying risk factors can offer some guidance, but they are not definitive predictors for PND onset. As such, midwives are advised to refer women displaying PND symptoms to mental health specialists for comprehensive evaluation (Boyd et al. 2005).

As mentioned before, stigma surrounding mental illness is driven primarily by two elements: cognitive understanding of the condition and emotional reactions, which may be rooted in prejudice (Thornicroft et al. 2007). Recognising this, we initiated the “MAternal Mood Assessment” (MAMA) study to gauge midwives’ knowledge and emotional attitudes toward PND, given their instrumental role in supporting women’s mental health throughout pregnancy and beyond.

## Materials and methods

The MAMA study is a cross-sectional analysis based on a voluntary, anonymous online survey carried out in January and February 2020. The survey was disseminated via social networks with a snowball technique through the channels of CiaoLapo (an Italian foundation dedicated to research and education on perinatal health) and of partner institutions. Participants voluntarily self-selected to complete the survey and they were considered eligible if they were midwives and worked with women during the perinatal period. Consent was provided at the start of the survey, once participants had read the participant information and met the eligibility criteria. No personal information was recorded; the language of the survey was Italian.

The survey was an online questionnaire with five parts: (A) participant’s background; (B) PND definition, treatment, and symptoms; (C) PND coping, communication, and support methods; (D) PND management opinions; and (E) PND training and experience. The survey, comprising 37 questions with either multiple-choice or open-ended responses, was designed by combining signs and symptoms from the DSM-5 (American Psychiatric Association 2013) with key risk factors identified in medical literature. Additional items were included to more accurately assess the midwives’ knowledge of PND. Further details on the instrument are available elsewhere (Ravaldi & Vannacci 2023a). Raw data of the survey and a full English translation of the questionnaire are available in an online repository (Ravaldi & Vannacci 2023b) (https://dx.doi.org/10.17632/sx23zmtcxv).

### Statistical analysis and data presentation

Survey responses were downloaded and extracted from the online survey tool Surveymonkey and imported into Excel for data management. Quantitative data were imported into Stata/BE 18.0 (StataCorp) for statistical analysis. Categorical data were reported as frequencies and percentages and compared using the chi-squared test, whereas continuous data were reported as mean values with standard deviations (SD) and compared using *t*-test. All results were considered to be statistically significant at *p* < 0.05. Figures were plotted using Tableau Desktop 2023.1 (Tableau Software, LLC).

## Results

### Sample characteristics

A total of 152 midwives, all women, took part in this study. Table 1 shows the demographic and professional characteristics of the sample. The majority of respondents (80.9%) took care of less than five mothers with PND during their career, with 32.9% having never assisted one of them. Only 24.3% of the responders had previously attended a specific formal training about PND.

**Table 1:** General characteristics of the sample

	***N* = 152 (%)**
**Age classes (years)**	
21.0–24.9	53 (34.9)
25.0–30.9	51 (33.6)
31.0–58.9	48 (31.6)
**Work years classes**	
0.0–0.9	59 (38.8)
2.0–2.9	26 (17.1)
4.0–8.9	29 (19.1)
10.0–34.9	38 (25.0)
**University clinic**	
No	138 (90.8)
Yes	14 (9.2)
**Delivery room**	
No	72 (47.4)
Yes	80 (52.6)
**Obstetric ward**	
No	74 (48.7)
Yes	78 (51.3)
**Pregnancy diseases**	
No	122 (80.3)
Yes	30 (19.7)
**Obstetric clinic**	
No	119 (78.3)
Yes	33 (21.7)
**Counselling service**	
No	138 (90.8)
Yes	14 (9.2)
**Private practice**	
No	139 (91.4)
Yes	13 (8.6)
**Currently not working**	
No	124 (81.6)
Yes	28 (18.4)
**Place of work—other**	
No	142 (93.4)
Yes	10 (6.6)
**Number of patients with post-natal depression**	
None	50 (32.9)
< 5	73 (48.0)
5–10	21 (13.8)
> 10	8 (5.3)
**Attended courses on perinatal mental health**	
Yes	37 (24.3)
No	111 (73.0)
Do not remember	4 (2.6)

### Midwives’ knowledge about PND

**Fig. 1:**
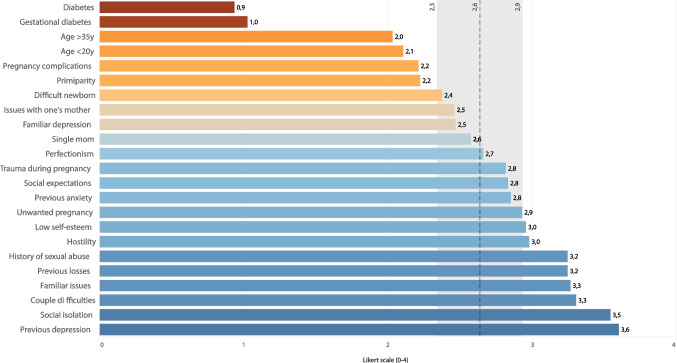
Possible risk factors for perinatal depressions

We asked midwives to rate the importance of various risk factors for PND on a Likert-type scale from 0 (“not important”) to 4 (“extremely important”). The risk factors that received the lowest ratings were as follows: diabetes before or during pregnancy, being younger than 20 or older than 35, having the first child, having difficulties in managing the newborn, and having experienced complications during pregnancy (Fig. 1).

**Fig. 2:**
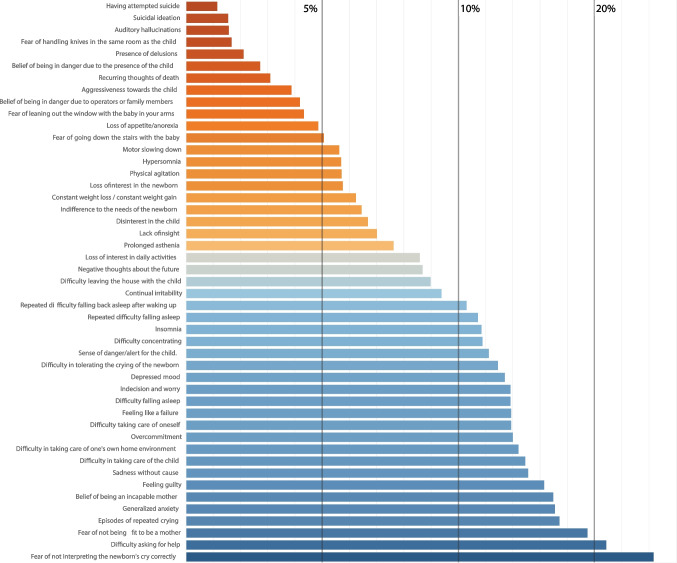
Frequency in the presentation of symptoms in clinical practice

Most midwives (91.5%) correctly defined PND as “the presence of a major depressive disorder during pregnancy or after birth”. However, only 36.9% of them correctly identified the third trimester of pregnancy as the most common time for PND to occur, while 34.8% chose the first or second trimester and 28.3% did not know. Regarding the onset of PND after childbirth, there was no clear consensus among midwives: 36.2% said it happened “in the first week”, 29.0% said “after 2 weeks”, and 32.2% said “within a year”. We also explored the symptoms that midwives encountered when caring for mothers with PND after childbirth using a Likert-type scale from (“never”) to (“more than 20% of women”). The most frequently reported symptoms were as follows: fear of not interpreting the newborn’s cry correctly, difficulty in asking for help, fear of not being fit to be a mother, episodes of repeated crying, anxiety, belief of being an incapable mother, and guilt. Severe symptoms such as suicide ideation or attempts, delusions, and hallucinations were rare, reported in less than 2% of cases (Fig. 2).

### PND clinical management

**Fig. 3:**
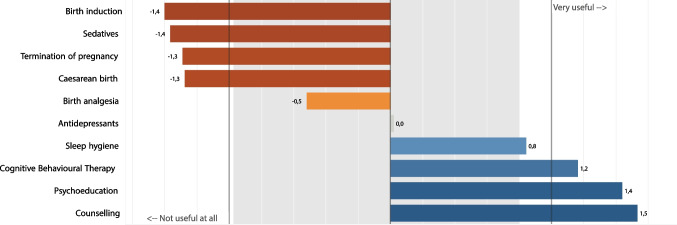
Most useful treatments and approaches

Sixty-eight percent of midwives reported the lack of a specific pathway of care for PND in their workplace and almost all of them (99.3%) agreed that having guidelines about PND management would be very helpful. We also asked midwives to rate the usefulness of various treatments for PND during pregnancy on a Likert-type scale from 0 (“not at all useful”) to 4 (“very useful”). The treatments that received lowest ratings by most midwives were labour induction (100%), sedation during childbirth (96.0%), ToP (94.7%), C-Sect. (98.5%), and birth analgesia (90.1%). Only 25.7% of midwives considered psychotropic medication as “useful” or “very useful” aids, while most of them rated sleep hygiene (65.8%), cognitive and behavioural therapy (78.3%), psychoeducation (89.5%), and counselling (94.1%) as “useful” or “very useful” interventions for depression during pregnancy. Figure 3 shows mean scores for each treatment (range − 2/ + 2).

Midwives identified some other measures that could support women with PND symptoms during pregnancy, such as scheduling follow-up visits (77.0%), referring women to public outpatient services (73.7%), ensuring home care service (56.6%), and informing relatives about PND symptoms (43.4%). Only a minority of midwives (22.4%) thought that referring mothers to a perinatal psychiatric service would be useful.

About breastfeeding, most midwives (91%) thought that it was not only safe but also advisable if the mother wanted it. However, 24.2% of midwives thought that breastfeeding was “not possible” and 25.8% thought that it was “hardly possible” if the mother was taking psychotropic medication.

#### Management when PND is suspected

We also explored what midwives usually do when PND is suspected. Almost all midwives agreed that it was better to support patients immediately talking with a psychologist about the situation (95.5%); 34% of them thought that referring patients to a psychiatrist was appropriate; 55.3% of midwives thought that talking with patients’ relatives could be helpful. We asked midwives which professionals were the most suitable to support women with a diagnosis of PND. The most frequently chosen were psychologists (85.5%) and midwives (78.9%), followed by psychiatrists (32.9%), general practitioners (31.6%), and obstetricians (17.8%). In addition, midwives reported that they usually discussed cases of PND with their colleagues (other midwives) (64.5%) or with psychologists (43.4%), while gynaecologists (24.3%) and psychiatrists (9.2%) were less involved. Moreover, our results pointed out that there is a lack of a formal pathway of care between hospital and public outpatient care services. Only 35.6% of midwives were certain about which outpatient care services were available for mothers with PND.

#### Communication and relational skills

We also assessed midwives’ communication and relational skills implemented with mothers with a suspected PND and their relatives. The sample reported that the most suitable ways to establish a relationship with mothers were as follows: listening to their needs (79.6%), ensuring continuity of care (79.6%), providing the opportunity to talk (73.0%), showing respect for their mental health condition (72.4%), and showing empathy (65.8%); 80.3% of midwives preferred open questions as the best technique to build a relationship with mothers with a suspected PND.

Relatives were involved in mothers’ care by communicating the diagnosis to the woman first, then deciding with her which family member should be informed (66.4%), and offering support to all family members (49.3%). In addition, most midwives (83.6%) believed that empathic fathers could be very helpful for women with PND symptoms.

### Midwives’ experience with PND

We also examined the experience of midwives with PND in their clinical practice; 52.6% of them reported that mothers do not ask for help. According to them, the main sources of support that mothers sought are as follows: birthing class meetings (15.1%), prenatal medical examinations (13.2%), and peer groups (19.7%).

Midwives also described the challenges of talking to mothers with PND. The most common feelings that they experienced in this situation were as follows: fear of saying (63.8%) or doing (32.9%) the wrong thing, tenderness (33.6%), and concern for the mother’s future (57.2%). Moreover, they had mixed feelings when they learned that a patient had PND: 30.2% of them felt bad for having overlooked some signs, 20.3% felt inadequate, and 17.8% empathised and identified with the mothers. Only a small percentage of the sample (2.0%) usually distanced themselves from the situation and did not feel emotionally involved in PND treatment.

When midwives’ answers were checked against current literature on PND, only 36 of them (23.7%) were classified as having a good knowledge of the disorder. These midwives reported significantly less fear of saying the wrong things to mothers (25.8% vs 74.2%, *p* = 0.006) and were less worried for the mother’s future (38.9% vs 62.95%, *p* = 0.01).

The detailed answers for all explored areas, as well as criteria for classifying PND knowledge, are reported in **Table S1**.

## Discussion

Midwives work in different contexts and settings, which makes it hard for them to recognise and manage PND. A study by the Italian Institute of Health found that there are about 1800 outpatient care centres where midwives offer prenatal and postnatal care to women who are pregnant or have given birth. But not all of these centres have specific pathways for perinatal depression (EpiCentro 2019).

MAMA study showed that only 23.7% of midwives had a good knowledge of PND. This is consistent with previous international studies, as shown in an integrative review of 2018, that have reported low levels of knowledge and confidence among midwives in dealing with this disorder (Noonan et al. 2018). Our findings indicate that midwives more knowledgeable in PND exhibited less fear of miscommunication and concern for maternal outcomes. This relationship suggests that an enhanced understanding of PND can positively affect midwives’ communication and emotional responses, which are critical in early detection and referral of PND cases. Carroll et al. (2018) highlight the importance of healthcare professionals’ communication skills in managing maternal mental health, implying that midwives with better PND knowledge might be more effective in navigating these complex interactions (Carroll et al. 2018). Thus, enhancing midwives’ PND knowledge may not only increase their proficiency but also their self-efficacy and empathy in delivering perinatal mental health care (Shinohara et al. 2022).

Recognising perinatal depression is a complex task for healthcare professionals (HCPs) and mothers, largely due to its nuanced symptoms. For example, “baby blues”, changes in appetite, and sleep disturbances during prenatal and postnatal periods can often obfuscate an accurate diagnosis. Specifically, “baby blues” is a condition that affects a majority of women postpartum, yet its symptoms generally dissipate within 2 weeks. Intriguingly, only one-third of the midwives in our sample correctly identified this 2-week threshold that differentiates “baby blues” from perinatal depression (Beck 2006; Kroh & Lim 2021). Our results offer a dual perspective: on one side, the midwives demonstrated theoretical knowledge of some prevalent symptoms of perinatal depression, indicating an ability to identify women at higher risk. On the flip side, they overlooked other symptoms that are commonly documented in the current scientific literature (Leigh & Milgrom 2008; Milgrom et al. 2008; Yang et al. 2022). Furthermore, midwives reported encountering severe symptoms such as neglecting the newborn’s needs or having suicidal thoughts less frequently than one might expect based on existing studies (Beck 2006; Kim et al. 2015; Ugarriza DN 2002). This suggests that there may be gaps in their understanding of key features of perinatal depression, making it difficult for them to accurately identify and manage this condition.

Women often hesitate to discuss their emotional well-being with healthcare professionals due to societal stigma, fear of being labelled as “bad mothers”, and negative experience with the healthcare system which inhibits their help-seeking behaviour and hampers effective treatment (Barney et al. 2006; Buist et al. 2005, 2007; Button et al. 2017). Our study corroborates this reluctance among mothers to seek help and underscores the complexity of diagnosing perinatal depression due to varying definitions and guidelines among the different professionals involved in care (AAP, Acog., 2017; ACOG 2015). Formal training aimed at enhancing midwives’ understanding of perinatal depression could reduce such stigmatising attitudes (Li et al. 2015). Although HCPs recognise the critical importance of early diagnosis, they also find it challenging to achieve.

Interestingly, midwives showed a preference for referring mothers to psychologists over psychiatrists. This tendency could stem from a stigma toward mental health practitioners and limited knowledge about psychological and psychiatric services. In Italy, the absence of a formalised care pathway for suspected PND and the lack of guaranteed access to psychotherapy services in the National Health System, which is the first-line treatment (Palumbo et al. 2016), might influence these referral choices. Referring to psychiatrists, who are legally authorised to provide psychotherapy in Italy, could standardise the PND care pathway, offering immediate support to mothers.

Further complicating matters, our data revealed a divided opinion among midwives regarding the efficacy of psychotropic medication. Nearly half expressed concerns regarding the effectiveness of antidepressants in PND treatment, while the other half recognised their importance. The use of antidepressants can be an important component of PND treatment, especially for more severe cases. While research indicates mixed evidence regarding the specific efficacy of antidepressants in the perinatal period, it is likely that positive effects found with antidepressants in the general depression literature would translate to depressed postnatal women as well. When considering antidepressant use for breastfeeding mothers with PND, studies suggest it is generally viewed as a reasonable treatment option despite the lack of definitive research on effects of exposure in breastfed infants. Within a multidisciplinary care context, antidepressants may provide an effective option as part of an overall support plan for mothers suffering from PND (O’Hara et al. 2015).

Establishing a strong relationship and effective communication is pivotal for supporting women and facilitating the early detection of perinatal depression. The process of screening for this condition demands sensitivity, as it involves a nuanced dialogue between the mother and a trusted HCP, which can significantly influence the mother’s receptivity to the screening itself (El-Den et al. 2015). The rapport between midwives and mothers is crucial yet complex, often fraught with challenges. Common concerns among midwives include feelings of inadequacy and the fear of making a verbal misstep. To address these issues, it is imperative to bolster midwives’ communication and relational skills. By cultivating a trusting and empathic bond with mothers, midwives can more effectively identify and delve into the emotional concerns and vulnerabilities that women might otherwise keep hidden or disregard. This, in turn, enhances the likelihood of recognising risk factors and frailties that may go unnoticed.

Regarding breastfeeding, a significant majority of the surveyed midwives believed it to be a viable option for mothers with perinatal depression, provided the mothers themselves desired it. However, nearly half of the respondents felt that breastfeeding would be inadvisable for mothers taking medication for PND. This viewpoint contrasts with existing literature, which emphasises the manifold benefits of breastfeeding for both mothers and infants, including enhancing maternal self-efficacy and serving as a protective factor against postpartum depression (Tucker & O’Malley 2022). Furthermore, effective screening for PND could reduce rates of breastfeeding cessation by connecting mothers with appropriate support services.

In our survey, the continuity of care stood out as a pivotal issue. A significant portion of the midwives surveyed were unaware of the available outpatient support services, while an equally large group cited a lack of established care pathways. Similar to diagnostic challenges, the data indicate that while midwives may understand the clinical steps involved in managing perinatal depression, they often struggle with the actual implementation of a care plan. Additionally, midwives expressed difficulties in quickly locating “the right support” for patients, emphasising the risk of losing track of them. It becomes clear that ensuring continuity of care is unattainable without directing women to a multidisciplinary team that includes specialists such as psychiatrists, gynaecologists, paediatricians, and psychologists (Paschetta et al. 2014).

In conclusion, effective screening and collaborative, multidisciplinary care between midwives and mental health professionals, such as psychologists and psychiatrists, were identified as critical factors that could significantly improve outcomes for mothers with perinatal depression. Given the limitations of existing screening tools, conducting a comprehensive psychiatric history assessment that specifically evaluates risk factors for PND appears to be the most effective approach. As previously emphasised, midwives serve a pivotal role in identifying women at risk for PND, since their frequent interactions with mothers uniquely position them to detect shifts in mental health status, further underscoring their importance in the diagnostic and management process (Gerber 2019; Hoffman 2021).

## Strengths and limitations

One of the major strengths of this study is that it is the first research with the aim of exploring Italian midwives’ knowledge and attitudes toward perinatal depression, offering an expansive view of various facets of perinatal mental healthcare. This provides valuable insights into the specific needs midwives have in identifying and supporting women with this condition. However, it is essential to acknowledge some limitations. The study’s primary constraint is its focus solely on midwives, excluding other healthcare professionals, and omitting certain risk factors like preterm birth from evaluation. This exclusion was deliberate, as this research serves as an inaugural study in this specific area. Moreover, the study’s cohort consists of self-selected midwives, possibly not representing the entire spectrum of Italian midwives. Notably, a significant proportion were hospital midwives, which may not fully encompass the varied roles of territorial midwives in Family Service Units who engage extensively in awareness, education, prevention, diagnosis, and treatment activities in perinatal care. Another constraint is the dependence on self-reported data, which may be subject to biases such as social desirability and recall inaccuracies.

## Conclusions

In light of the profound impact that perinatal depression can have on both mothers and newborns, early intervention is not just beneficial—it is imperative. This study has highlighted concerning gaps in midwives’ understanding of perinatal depression, from symptom recognition to risk factors, potentially exacerbating the stigma surrounding perinatal mental health. Our findings underscore the urgent need for formal training programmes during midwives’ university studies that not only enhance diagnostic accuracy but also confront and dispel stigmatising attitudes since the beginning of their career. Creating a non-judgmental environment, ensuring positive interactions within healthcare systems, and providing accurate information about PND symptoms are crucial in enhancing mothers’ willingness to seek help. As this is the inaugural study exploring the perspectives of Italian midwives on this critical issue, further research involving a wider range of healthcare professionals in the obstetric field is essential to develop a more comprehensive understanding and to foster a continuity of care and a multidisciplinary approach to PND.

## References

[CR1] AAP ACOG (2017) Guidelines for perinatal care (8th ed.). American Academy of Pediatrics, American College of Obstetricians and Gynecologists

[CR2] ACOG (2015). The American College of Obstetricians and Gynecologists Committee Opinion no. 630. Screening for perinatal depression. Obstet Gynecol.

[CR3] ACOG (2023). Screening and diagnosis of mental health conditions during pregnancy and postpartum: ACOG clinical practice guideline No. 4. Obstet Gynecol.

[CR4] American Psychiatric Association (2013). Depressive disorders. Diagnostic and statistical manual of mental disorders.

[CR5] Barney LJ, Griffiths KM, Jorm AF, Christensen H (2006). Stigma about depression and its impact on help-seeking intentions. Aust NZ J Psychiat.

[CR6] Beck CT (2006). Postpartum depression: it isn’t just the blues. Am J Nurs.

[CR7] Boyd RC, Le HN, Somberg R (2005). Review of screening instruments for postpartum depression. Arch Women Ment Hlth.

[CR8] Buist A, Bilszta J, Barnett B, Milgrom J, Ericksen J, Condon J, Hayes B, Brooks J (2005). Recognition and management of perinatal depression in general practice—a survey of GPs and postnatal women. Aust Fam Physician.

[CR9] Buist A, Ellwood D, Brooks J, Milgrom J, Hayes BA, Sved-Williams A, Barnett B, Karatas J, Bilszta J (2007). National program for depression associated with childbirth: the Australian experience. Best Pract Res Clin Obstet Gynaecol.

[CR10] Button S, Thornton A, Lee S, Shakespeare J, Ayers S (2017). Seeking help for perinatal psychological distress: a meta-synthesis of women’s experiences. Br J Gen Pract.

[CR11] Carroll M, Downes C, Gill A, Monahan M, Nagle U, Madden D, Higgins A (2018). Knowledge, confidence, skills and practices among midwives in the Republic of Ireland in relation to perinatal mental health care: the mind mothers study. Midwifery.

[CR12] Cena L, Mirabella F, Palumbo G, Gigantesco A, Trainini A, Stefana A (2021). Prevalence of maternal antenatal and postnatal depression and their association with sociodemographic and socioeconomic factors: a multicentre study in Italy. J Affect Disord.

[CR13] Cornally N, McCarthy G (2011). Help-seeking behaviour: a concept analysis. Int J Nurs Pract.

[CR14] Cox JL, Holden JM, Sagovsky R (1987). Detection of postnatal depression. Development of the 10-item Edinburgh Postnatal Depression Scale. Br J Psychiatry.

[CR15] Earls MF, T Committee on Psychosocial Aspects of Child and Family Health (2010) Incorporating recognition and management of perinatal and postpartum depression into pediatric practice. Pediatrics 126(5):1032–1039. 10.1542/peds.2010-2348 10.1542/peds.2010-2348 20974776

[CR16] Eastwood JG, Jalaludin BB, Kemp LA, Phung HN, Barnett BEW (2012). Relationship of postnatal depressive symptoms to infant temperament, maternal expectations, social support and other potential risk factors: findings from a large Australian cross-sectional study. BMC Pregnancy Childbirth.

[CR17] El-Den S, O’Reilly CL, Chen TF (2015). A systematic review on the acceptability of perinatal depression screening. J Affect Disord.

[CR18] El-Den S, Pham L, Anderson I, Yang S, Moles RJ, O’Reilly CL, Boyce P, Raine KH, Raynes-Greenow C (2022). Perinatal depression screening: a systematic review of recommendations from member countries of the Organisation for Economic Co-operation and Development (OECD). Arch Womens Ment Health.

[CR19] EpiCentro. (2019). Consultori familiari, la prima fotografia dell’ISS. https://www.epicentro.iss.it/consultori/indagine-2018-2019

[CR20] Escribà-Agüir V, Artazcoz L (2011). Gender differences in postpartum depression: a longitudinal cohort study. J Epidemiol Community Health.

[CR21] Faisal-Cury A, Levy RB, Azeredo CM, Matijasevich A (2021). Prevalence and associated risk factors of prenatal depression underdiagnosis: a population-based study. Int J Gynaecol Obstet.

[CR22] Gaillard A, Le Strat Y, Mandelbrot L, Keïta H, Dubertret C (2014). Predictors of postpartum depression: prospective study of 264 women followed during pregnancy and postpartum. Psychiatry Res.

[CR23] Gavin NI, Gaynes BN, Lohr KN, Meltzer-Brody S, Gartlehner G, Swinson T (2005). Perinatal depression: a systematic review of prevalence and incidence. Obstet Gynecol.

[CR24] M Gerber 2019 Trauma-informed maternity care In Trauma-informed healthcare approaches Springer, New York

[CR25] Grekin R, Thomas EBK, Miller ML, O’Hara MW (2022). The role of prenatal posttraumatic stress symptoms among trauma exposed women in predicting postpartum depression. Stress Health.

[CR26] Hauck YL, Kelly G, Dragovic M, Butt J, Whittaker P, Badcock JC (2015). Australian midwives knowledge, attitude and perceived learning needs around perinatal mental health. Midwifery.

[CR27] Henderson C, Noblett J, Parke H, Clement S, Caffrey A, Gale-Grant O, Schulze B, Druss B, Thornicroft G (2014). Mental health-related stigma in health care and mental health-care settings. Lancet Psychiatry.

[CR28] Hoffman MC (2021). Pushing beyond the silos: the obstetrician’s role in perinatal depression care. J Matern Fetal Neonatal Med.

[CR29] Decreto Interministeriale 2 aprile 2001—Determinazione delle classi delle lauree universitarie delle professioni sanitarie, (2001)

[CR30] Kim JJ, La Porte LM, Saleh MP, Allweiss S, Adams MG, Zhou Y, Silver RK (2015). Suicide risk among perinatal women who report thoughts of self-harm on depression screens. Obstet Gynecol.

[CR31] Knaak S, Mantler E, Szeto A (2017). Mental illness-related stigma in healthcare: barriers to access and care and evidence-based solutions. Healthc Manage Forum.

[CR32] Leigh B, Milgrom J (2008). Risk factors for antenatal depression, postnatal depression and parenting stress. BMC Psychiatry.

[CR33] Li J, Li J, Thornicroft G, Yang H, Chen W, Huang Y (2015). Training community mental health staff in Guangzhou, China: evaluation of the effect of a new training model. BMC Psychiatry.

[CR34] Lim G (2021). Perinatal depression. Curr Opin Anaesthesiol.

[CR35] Martin CJH, Norris G, Martin CR (2020). Midwives’ role in screening for antenatal depression and postnatal depression. Brit J Midwifery.

[CR36] Matthey S, Agostini F (2017). Using the Edinburgh Postnatal Depression Scale for women and men-some cautionary thoughts. Arch Womens Ment Health.

[CR37] Matthey S, Henshaw C, Elliott S, Barnett B (2006). Variability in use of cut-off scores and formats on the Edinburgh Postnatal Depression Scale: implications for clinical and research practice. Arch Womens Ment Health.

[CR38] Mccauley K, Elsom S, Muir-Cochrane E, Lyneham J (2011). Midwives and assessment of perinatal mental health. J Psychiatr Ment Health Nurs.

[CR39] McGrath JM, Records K, Rice M (2008). Maternal depression and infant temperament characteristics. Infant Behav Dev.

[CR40] Mcloughlin J (2013). Stigma associated with postnatal depression: a literature review. Brit J Midwifery.

[CR41] Milgrom J, Gemmill AW, Bilszta JL, Hayes B, Barnett B, Brooks J, Ericksen J, Ellwood D, Buist A (2008). Antenatal risk factors for postnatal depression: a large prospective study. J Affect Disord.

[CR42] Moore Simas TA, Whelan A, Byatt N (2023). Postpartum depression—new screening recommendations and treatments. JAMA.

[CR43] Noonan M, Doody O, Jomeen J, Galvin R (2017). Midwives’ perceptions and experiences of caring for women who experience perinatal mental health problems: an integrative review. Midwifery.

[CR44] Noonan M, Jomeen J, Galvin R, Doody O (2018). Survey of midwives’ perinatal mental health knowledge, confidence, attitudes and learning needs. Women Birth.

[CR45] O’Hara MW, Dennis C-L, McCabe JE, Galbally M (2015). Evidence-based treatments and pathways to care. Identifying perinatal depression and anxiety: evidence-based practice in screening, psychosocial assessment, and management.

[CR46] Palumbo G, Mirabella F, Cascavilla I, Del Re D, Romano G & Gigantesco A (2016) Rapporto ISTISAN 16|31—Prevenzione e intervento precoce per il rischio di depressione post partum. Istituto Superiore di Sanità

[CR47] Park S-H, Kim J-I (2022). Predictive validity of the Edinburgh postnatal depression scale and other tools for screening depression in pregnant and postpartum women: a systematic review and meta-analysis. Arch Gynecol Obstet.

[CR48] Paschetta E, Berrisford G, Coccia F, Whitmore J, Wood AG, Pretlove S, Ismail KMK (2014). Perinatal psychiatric disorders: an overview. Am J Obstet Gynecol.

[CR49] Räisänen S, Lehto SM, Nielsen HS, Gissler M, Kramer MR, Heinonen S (2014). Risk factors for and perinatal outcomes of major depression during pregnancy: a population-based analysis during 2002–2010 in Finland. BMJ Open.

[CR50] Ravaldi C, & Vannacci A (2023b) MAMA: MAternal Mood Assessment survey for healthcare professionals Mendeley 10.17632/SX23ZMTCXV.1

[CR51] Ravaldi C, Vannacci A (2023). Data of the MAternal Mood Assessment (MAMA) survey for healthcare professionals: a pilot study on midwives in Italy. Data Brief.

[CR52] Schmidt E-M, Décieux F, Zartler U, Schnor C (2023). What makes a good mother? Two decades of research reflecting social norms of motherhood. J Fam Theor Rev.

[CR53] Shinohara E, Ohashi Y, Hada A, Usui Y (2022). Effects of 1-day e-learning education on perinatal psychological support skills among midwives and perinatal healthcare workers in Japan: a randomised controlled study. BMC Psychol.

[CR54] Thornicroft G, Rose D, Kassam A, Sartorius N (2007). Stigma: ignorance, prejudice or discrimination?. Br J Psychiatry.

[CR55] Thornicroft G, Mehta N, Clement S, Evans-Lacko S, Doherty M, Rose D, Koschorke M, Shidhaye R, O’Reilly C, Henderson C (2016). Evidence for effective interventions to reduce mental-health-related stigma and discrimination. Lancet.

[CR56] Tucker Z, O’Malley C (2022). Mental health benefits of breastfeeding: a literature review. Cureus.

[CR57] Ugarriza, DN (2002) Postpartum depressed women’s explanation of depression. J Nurs Scholarship 34(3):227–233. 10.1111/j.1547-5069.2002.00227.x 10.1111/j.1547-5069.2002.00227.x 12237984

[CR58] Woody CA, Ferrari AJ, Siskind DJ, Whiteford HA, Harris MG (2017). A systematic review and meta-regression of the prevalence and incidence of perinatal depression. J Affect Disord.

[CR59] Yağmur Y, Ulukoca N (2010). Social support and postpartum depression in low-socioeconomic level postpartum women in Eastern Turkey. Int J Public Health.

[CR60] Yang K, Wu J, Chen X (2022). Risk factors of perinatal depression in women: a systematic review and meta-analysis. BMC Psychiatry.

